# Comparing the effect of intravenous versus intracranial grafting of mesenchymal stem cells against parkinsonism in a rat model: Behavioral, biochemical, pathological and immunohistochemical studies

**DOI:** 10.1371/journal.pone.0296297

**Published:** 2024-02-13

**Authors:** Amina Essawy Essawy, Oryhan Aly Abou-ElNaga, Radwa Ali Mehanna, Noha Mohammed Badae, Eman Sheta Elsawy, Ahmed Abdelmagied Soffar

**Affiliations:** 1 Department of Zoology, Faculty of Science, Alexandria University, Alexandria, Egypt; 2 Department of Physiology, Faculty of Medicine, Alexandria University, Alexandria, Egypt; 3 Department of Pathology, Faculty of Medicine, Alexandria University, Alexandria, Egypt; Federal University of Ceara: Universidade Federal do Ceara, BRAZIL

## Abstract

Parkinson’s disease (PD) is one of the most common neurodegenerative diseases worldwide. Currently applied therapeutic protocols are limited to improve the motor functions of patients. Therefore, seeking alternative regimes with better therapeutic impact is crucial. This study aims to validate the therapeutic impact of mesenchymal stem cell injection using two delivery methods, intracranial administration and intravenous administration, on rotenone (ROT)-induced PD model in rats. Our work included behavioral, biochemical, histological, and molecular investigations. Open field test (OFT) and rotarod tests were applied. Important oxidative stress, antioxidant and proinflammatory markers were monitored. Substantia Nigra and Striatum tissues were examined histologically and the molecular expression of DOPA decarboxylase, Tyrosine hydroxylase, and α-synuclein in neurons in these tissues were investigated. Our results showed that MSC grafting improved motor and memory impairments and oxidative stress status that were observed after ROT administration. Additionally, BM-MSCs application restored SOD and CAT activities and the levels of DA, L-Dopa, IL6, IL1β, and TNFα. Moreover, MSC grafting overwhelmed the pathological changes induced by ROT and normalized the expression of Tyrosine hydroxylase, DOPA decarboxylase, and α-synuclein towards the control values in the Nigral and Striatal tissues of male rats. Conclusively, both administration routes improved motor function, protection of the nigrostriatal system, and improved striatal dopamine release. The observed beneficial effect of applying MSCs suggests potential benefits in clinical applications. No significant differences in the outcomes of the treatment would favor a certain way of MSC application over the other. However, the intravenous delivery method seems to be safer and more feasible compared to the intrastriatal method.

## Introduction

Parkinson’s disease (PD) is the most common synucleinopathy and one of the most prevalent neurodegenerative diseases worldwide. The primary cause of this disease is a deficiency of dopamine in the basal ganglia of the brain. Dopamine deficiency results from the specific loss of DAergic neurons in the Substantia Nigra (SN), Striatum (ST), locus coeruleus, dorsal vagal nucleus, and cerebral cortex. A characteristic pathologic hallmark of PD is the presence of neuronal intracytoplasmic alpha-synuclein inclusions, known as Lewy bodies, in dopamine-producing areas within the brain [1, 2]. The precise mechanism that leads to neuronal death in PD is not fully understood, but it appears to be multifactorial and involves inflammation, oxidative stress, genetic factors, glial dysfunction, excitotoxicity, and other factors [3, 4].

Up to date, there is no cure for PD [5]. Currently applied therapeutic options do not alter the progression of the disease and do not alleviate the non-motor symptoms of PD, but rather improve the motor functions of the majority of patients, especially at early stages of the disease [6]. Therefore, seeking alternative therapies with better therapeutic impact is crucial for PD patients.

Mesenchymal stem cells (MSCs) are multipotent progenitor cells with a differentiation capacity to form other types of cells such as neurons, hepatocytes, cartilage, adipocytes, etc. [7]. In addition, MSCs are able to secrete several neurotrophic factors, modulate inflammation, and possibly act as mitochondrial donors [8–10]. Due to the aforementioned characteristics, MSCs have received increasing attention with regard to their potential use as cell therapy in different medical conditions, including neurological diseases [11, 12].

There are two possible ways for bone marrow-derived MSC transplantation: intrastriatal injection or intravenous injection. Both ways successfully deliver MSCs to the site of damage and may contribute to improving motor functions. However, whether a certain method of administration is more efficient than the other is still questionable. To date, the previously published literature has not addressed this question. Therefore, this study aims to validate the therapeutic impact of MSC injection using the previously mentioned routes of delivery on a rotenone-induced PD model in rats. Our study includes behavioral, physiological, histological, and molecular studies. Additionally, we will compare the potential benefits of both administration routes to determine if a certain technique provides better therapeutic outcomes.

## Material and methods

### Experimental animals

The experiment was performed using fifty-six adult male Wistar Albino rats (three months old) with an average body weight of 200 g. Males were chosen in this work for their more stable hormonal profile compared to females. Two additional younger rats aged three weeks were used as a source of the MSCs. Animals were purchased from Experimental Animal House in Medical Physiology Department, Faculty of Medicine, Alexandria University, and were kept in standard stainless-steel cages at room temperature 25–28°C, under standard conditions of a 12 h light-dark cycle. The rats were fed a standard pellet diet and water *ad libitum* and allowed to acclimatize for 1 week prior to experimentation.

All experimental procedures and animal handling were conducted in strict accordance with the guidelines approved by Alexandria University Institutional Animal Care and Use Committee (ALEXU-IACUC), a member of the International Council for Laboratory Animal Science (ICLAS), and in accordance with ARRIVE guidelines. The ethical approval number for this work is AU04190824201.

### Chemicals

All reagents and chemicals used were of high analytical grade. Rotenone (Purity, 98%) was purchased from Sigma-Aldrich (St Louis, MO, USA). Double-distilled water was used as the solvent. Nitric oxide, lipid peroxide, glutathione, catalase, and superoxide dismutase standard commercial kits were purchased from Biodiagnostic Co. (Cairo, Egypt). Rat interleukin-6 (IL-6) enzyme-linked immunosorbent assay kit was purchased from (Boster Picokine™ biological technology Co., (Pleasanton). ELISA kit for tumor necrosis factor-alpha (TNF-α) was purchased from (R&D Systems®, Inc., USA), Rat l-3,4-dihydroxyphenylalanine (L-DOPA) was purchased from (MyBioSource CO., Inc., San Diego, USA), Rat interleukin-1β (IL-1β) (BioVendor research and diagnostic product CO., Brno, Czech Republic, Germany), and Dopamine (DA) purchased from (Abnova CO., USA). Antibody kits for DOPA decarboxylase, tyrosine hydroxylase and alpha synuclein purchased from (Affinity Bioscience CO., USA). Other chemicals were of the highest purity commercially available.

### Experimental groups

Animals were divided randomly into control and experimental groups and treated as follows:

Group 1 (n = 6) **(Control):** Animals were injected subcutaneously with 1ml of sterilized saline daily for four weeks.

Group 2 (n = 50) (**Experimental group; Induced parkinsonism group**): Animals were subcutaneously injected with rotenone (2 mg/kg) **‎**daily for four weeks [13]. Animals in this group were divided into five subgroups, each containing ten rats, and were treated as follows:‎

- **PD Model group (n = 10) (Rotenone only):** The rats were treated with rotenone only.
- **Transplanted group-Intrastriatal (n = 10)**: Animals were treated with rotenone and injected intrastriataly with BM-MSCs (1×10^4^ cells/5μl complete culture medium (CCM)) [14].
- **Transplanted group-Intravenous (n = 10)**: Animals were treated with rotenone and injected intravenously with BM-MSCs (1 × 10^6^ cells/ 1 ml CCM) [15].
- **Sham-grafted-Intrastriatal (n = 10)**: Animals were treated with rotenone and injected (Stereotactic guided) intrastriataly with 5μl CCM.
- **Sham-grafted-Intravenous (n = 10)**: Animals were treated with rotenone and injected intravenously with 1 ml CCM.

### Experimental procedure

#### Induction of parkinsonism

Parkinsonism was induced in 50 animals by subcutaneously injecting rotenone at a dose of 2 mg/kg body weight in 1 ml of a vehicle solution daily for four weeks [16].

#### Isolation, culture, and characterization of bone marrow-derived mesenchymal stem cells of male albino rat

The bone marrow of rats was harvested and cultured following the protocol described in Khalifa et al., 2019 [15]. Animals were decapitated under anesthesia and femurs and tibias were dissected out. Using sterile syringes, bones were flushed with sterile complete culture medium (CCM) (low glucose Dulbecco’s modified Eagle’s medium (LG-DMEM) (1.0 g/L glucose; Lonza, Switzerland) supplemented with 10% fetal bovine serum (FBS, HyClone, Biowest, France), 2 mM L-glutamine, and 1% Penicillin/Streptomycin (P/S, 10.000 IU/ml/10.000 μg/ml, Lonza, Switzerland). Flushed bone marrow was then passed through a sterile 70-μm cell strainer to remove any bone debris or cell clumps. The prepared cell cultures were passaged in a ratio of 1:3 to achieve 80%–90% confluence using 0.025% trypsin-EDTA solution (170.000 U/l-200 mg/l, Lonza) until the 3^rd^ passage, and then cryopreserved in complete culture medium with 5% DMSO until transplantation. The cells were characterized using fluorescent-labeled monoclonal antibodies for the mesenchymal marker CD90 and the hematopoietic marker CD45 surface markers. The analysis was performed using a BD FACSCalibur flow cytometer equipped with Cell Quest software (Becton Dickinson, New Jersey, USA).

#### BM-MSCs grafting

The procedure of intrastriatal grafting was performed in a sealed chamber. Animals were anesthetized using intraperitoneal injection of ketamine 100 mg/kg and xylazine 5 mg/kg. For Intrastriatal transplantation, animals were fixed in a stereotaxic instrument (David kopf instrument, Germany). A small (3–4 mm) incision was made in the scalp. A burr hole was made in the bone 3 mm lateral to bregma with a dental drill. Rats have received a bilateral intrastriatal infusion of Dil-labeled MSCs or vehicle using a Hamilton syringe. The infusion rate was 1 μl/minute at the exact stereotaxic coordinates: 1mm anterior to bregma, 2.6 mm lateral to the midline and 5.0 mm below the surface of the skull according to the Atlas of Paxinos and Watson [17]. After injection, the needle was slowly withdrawn and the burr hole was then filled with Gel-foam and the wound was closed with interrupted surgical sutures [14, 18]. All transplantation procedures were performed under aseptic conditions.

Regarding the intravenous grafting, anesthetized animals were injected with Dil-labeled MSCs or vehicle was slowly into the tail vein. The needle was kept in the tail vein for 5 min after the injection to avoid regurgitation and then carefully withdrawn.

Operated animals were individually placed in a heated recovery chamber until recovered from anesthesia, Then, each group of rats was housed in a cage. After surgery, all rats received gentamycin (5 mg/kg, intraperitoneally) for 3 days to prevent sepsis, and meloxicam (1mg/kg, subcutaneously) for analgesia. Saline injections (500 μl, subcutaneously) continued until the animals became hydrated and regained their pre-surgery weight. Immunosuppressants were not used in any animal.

#### Behavioral study

The Open Field Test (OFT) and Rotarod tests were used to assess the animals’ behavior. Prior to testing, the animals were acclimated to the open field and rotarod device three times. The OFT was used to evaluate spontaneous performances of rats, such as motor activities, anxiety-like behaviors, and rearing activity. The test was performed in a dimly lit room in a free exploration open-field arena made of wood with opaque walls measuring 50 cm x 30 cm x 20 cm (length x width x height). The floor of the field was divided into 16 equally sized squares (4 x 4 squares) [19]. The latency time (in seconds) and the ambulation frequency were measured. The number of grooming events and the number of rears were also recorded manually using a video camera.

The Rotarod test was performed as previously described [20]. The animals’ time on the rotarod cylinder was measured using an accelerating speed (4 to 40 rpm) within three minutes, and the mean latency to fall off the rotarod was recorded in seconds. The cylinder apparatus was cleaned with a 70% ethanol solution before each trial, and testing was recorded manually using a video camera.

#### Brain dissection and processing

Six weeks after transplantation, rats were sacrificed under deep anesthesia with xylazine and ketamine. After craniotomy, the whole brain was removed and washed with ice-cold saline. The left ST and the left SN were processed for neurochemical assays (see biochemical analysis). While the right ST and the right SN of the same rat were fixed in formalin (10%) for histological and immunohistochemical studies.

#### Histological investigation of SN and ST tissues

Histological changes were analyzed by the given procedure [21, 22]. Briefly, fixed specimens of SN and ST regions were dehydrated and embedded in wax. The tissue blocks were cut at 5 μm thick and stained with Hematoxylin and Eosin (H&E).

#### TH, DDC, and α-Synuclein staining in SN and ST tissues

The paraffinized SN and ST regions from control and experimental rats were sectioned coronally for immunohistochemical ‎studies to assess the expression of tyrosine hydroxylase (TH), DOPA decarboxylase (DDC) and alpha-synuclein ‎inclusions. 4-μm sections were cut and mounted on positively charged slides. Avidin–biotin immune peroxidase-based IHC was performed using primary antibodies against Tyrosine Hydroxylase (#AF6113, 1:100, Affbiotech, Changzhou, China), DOPA Decarboxylase (#DF12386, 1:100, Affbiotech, Changzhou, China) and α-Synuclein (#AF6285, 1:100, Affbiotech, Changzhou, China). After labeling with the primary antibody, slides were further stained with rabbit polyclonal antibodies kits according to the manufacturer’s protocols‎‎ (Affinity Bioscience).

#### Biochemical analysis

Six weeks after transplantation, the rats were fasted overnight and sacrificed under anesthesia. Nigrostriatal tissue of four rats from each group was quickly removed, washed with cold normal saline, minced, and homogenized (10% w/v) in 4 ml ice-cold sucrose buffer (0.25M). The homogenate was centrifuged at 10,000 rpm for 20 minutes at 4°C. The supernatant was stored at -80°C for biochemical studies.

Lipid peroxidation was measured by estimating the concentrations of malondialdehyde (MDA) (in nmol/g tissue) using the method of Ohkawa et al. (1979) [23]. Nitric oxide (NO) was measured as micromoles (μmol/g tissue) following its reduction into nitrite according to the method of Khayyat et al. (2018) [24]. The level of glutathione (GSH) content (in mg/g tissue) was spectrophotometrically estimated according to the method of Beutler et al. (1963) [25]. The activity of superoxide dismutase (SOD) and catalase (CAT) (in U/g tissue) was estimated as previously described by Fossati et al. (1980) [26]. Quantitative measurements of brain interleukin-1β (IL-1β), interleukin-6 (IL-6), and tumor necrosis factor-alpha (TNF-α) were performed using ELISA-based kits according to the manufacturer’s protocols. Dopamine (DA) and L-Dopa levels (measured as ng/g brain tissue) were detected using a rat microplate ELISA assay kit following the manufacturer’s protocol.

### Statistical analysis

The data were input into IBM SPSS software package version 20.0 (IBM Corp., Armonk, NY) and analyzed. The normality of distribution was verified using the Kolmogorov-Smirnov test. Quantitative data were described using the mean and standard error, expressed as (Mean ± SE), and analyzed using a one-way ANOVA test for normally distributed quantitative variables to compare between more than two groups. Pairwise comparisons were performed using the Post Hoc test (Tukey). P value of less than 0.05 was considered statistically significant.

## Results

### Culturing, characterization, and tracing of bone marrow-derived mesenchymal stem cells

**Fig 1:**
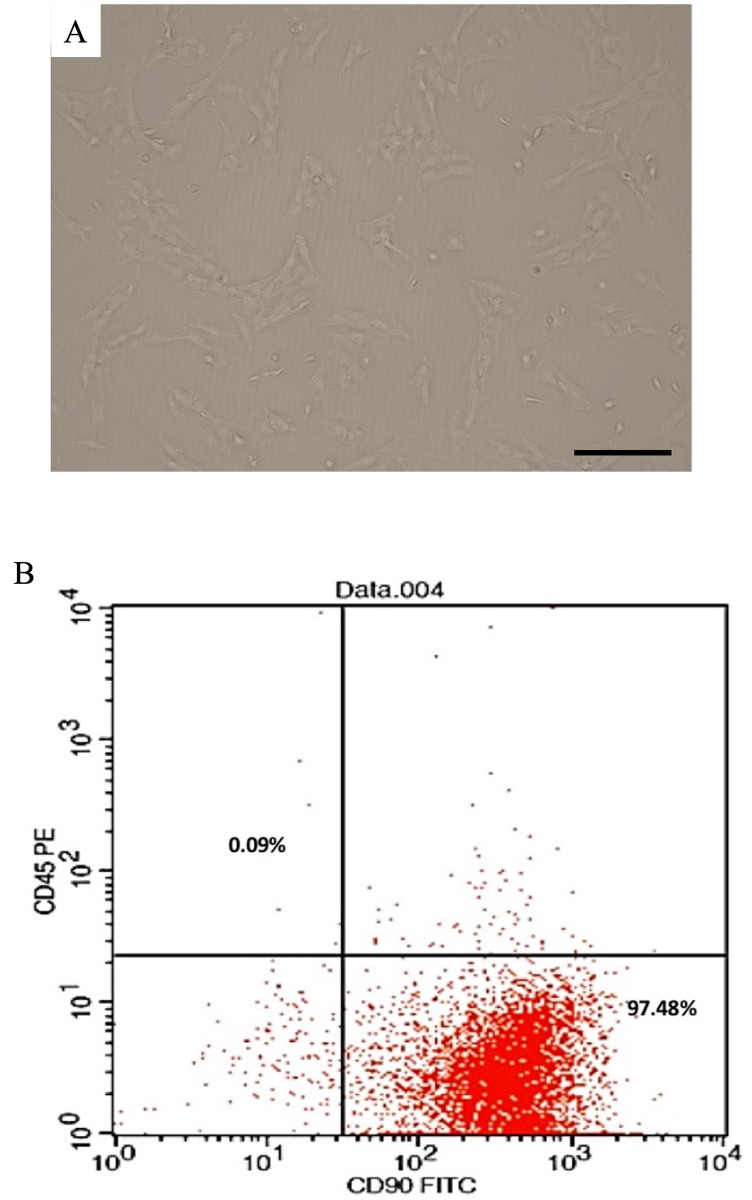
Characterization of bone marrow-derived mesenchymal stem cells. (A) Phase contrast images showing the morphology of BM-MSCs cultured at the third passage. (B) Flow cytometric analysis of cell-surface markers of BM-MSCs at passage 3. (Bar, 100 μm).

The isolated mesenchymal stem cells (MSCs) (Fig 1A) were stained against CD90 and CD45 and were analyzed using flow cytometry. Our results show that more than 99% of the population were expressing the CD90 marker (Fig 1B). On the other hand, these cells were CD45 negative.

### Stem cell administration ameliorates rotenone-induced PD behavioral abnormalities in rats

**Fig 2:**
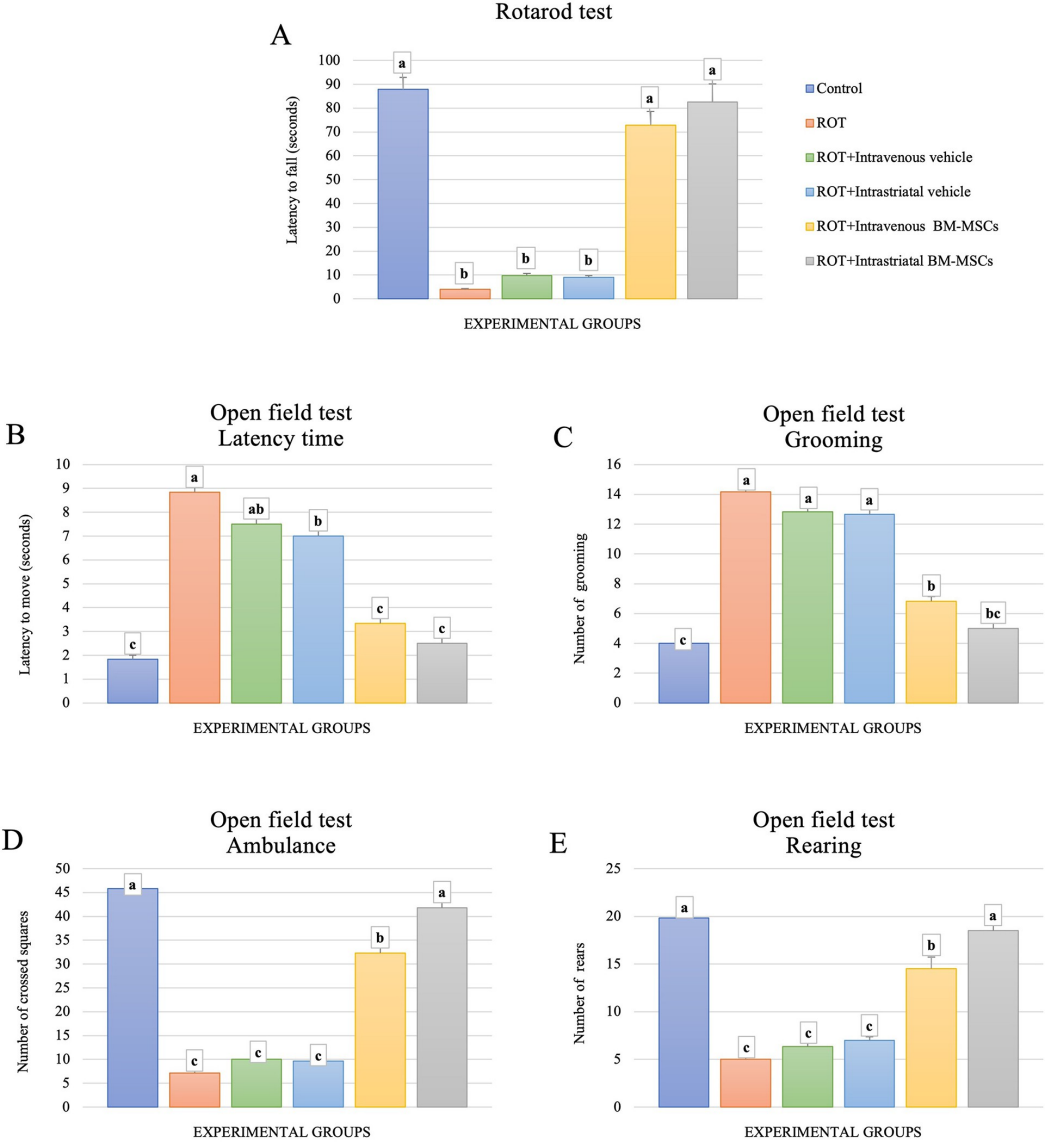
The impact of BM-MSCs on the behavioral alternation induced by rotenone in rats. (A) Rotarod test scores. (B-E) Open field test, measuring different parameters as indicated above the graphs. (ANOVA, n = 6, p < 0.05, Different letters indicate statistically significant differences).

On the rotarod apparatus, the effect of ROT and BM-MSCs treatment on the balance and coordination of male rats was investigated (Fig 2A). The mean latency to fall was significantly (p < 0.05) shorter in animals treated with ROT compared to the control group. Similar observations were recorded in the two groups of ROT-treated rats that were infused with vehicle intrastriataly or intravenously. On the other hand, the mean latency to fall was significantly (p < 0.05) increased in rats treated with BM-MSCs transplantation by two routes, intrastriatal and intravenous, compared to the ROT-treated group. However, rats treated with intrastriatal BM-MSCs administration showed longer latency to fall from the rotarod apparatus than those treated via the intravenous route. Additionally, ROT administration resulted in significant impairment in the locomotor and exploratory behaviors of animals in the open field test. This effect was demonstrated as a prolonged start moving latency, a decreased number of crossed squares, an increased exitance as the number of grooming increased, and a decreased number of rears. Upon administration of BM-MSCs (either intrastriatally or intravenously) to ROT-treated animals, the performance and locomotor functions of the animals were significantly improved. This effect was observed as a shortened latency time, an increased number of crossed squares, a decreased number of grooming, and an increased number of rears compared to the ROT-treated animals. The motor functions were slightly better when the BM-MSCs were applied via the intrastriatal route than in animals injected intravenously with MSCs. The results of the performed behavioral tests are presented in Fig 2B–2E.

### The Effect of stem cell transplantation on the histology of SN and ST

**Fig 3:**
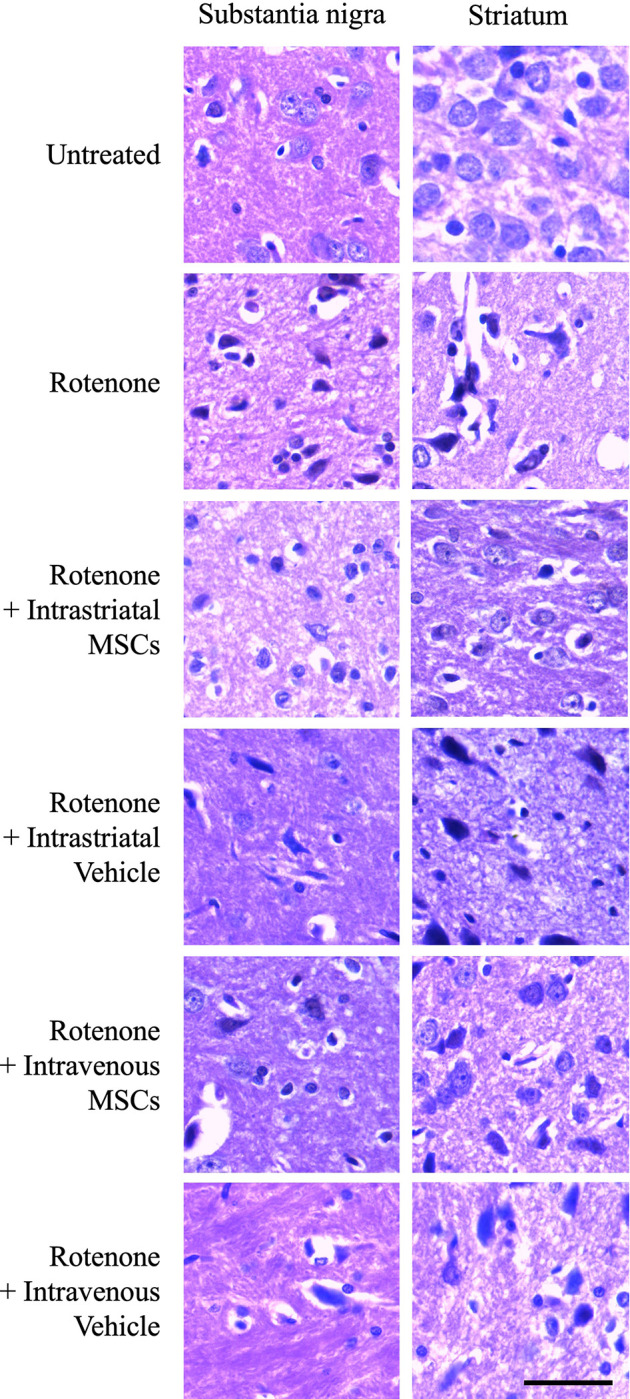
Representative images of brain sections showing the histological architecture of SN and ST in each group of animals. (Bar: 50 μm).

The normal histological appearance of Substantia Nigral tissue is shown in Fig 3 (Untreated group). Upon ROT treatment, there were dramatic degenerative changes (Fig 3, ROT and vehicle-treated groups). These alterations include nuclear irregularities, hyperchromasia, and pyknosis of some nuclei. The cytoplasm was deeply eosinophilic with wide peri-cellular hallos. Other changes include gliosis and perivascular edema. On the other hand, the SN tissues of the MSCs transplanted animals with intravenous route showed fewer neurons with hyperchromatic and pyknotic nuclei. When transplanting the MSCs intrastriatally using the Stereotaxis, the SN tissue appeared almost with normal structure. Representative images of the SN in the different experimental groups are shown in Fig 3.

Regarding the other DA-producing tissue, the ST, the normal histological structure of striatal tissue is shown in Fig 3 (Untreated group). The neurons appeared, with central rounded vesicular nuclei and small nucleoli, and were surrounded by a normal microglial population. Upon rotenone treatment, the ST tissue showed several degenerative features. The soma showed nuclear shrinkage, hyperchromasia, cytoplasmic eosinophilia, and vacuolization. Likewise, ST sections of both sham-grafted groups showed similar changes as appeared in the rotenone-treated animals. On the other hand, ST tissue of the MSCs transplanted group via intravenous route as well as intrastriatal route showed almost normal neuronal morphology and a lesser degree of degenerative changes. It is also worth to mention that the intrastriatal transplantation resulted in better pathological outcome with fewer alterations when compared with those observed in the intravenous transplantation animals.

### MSC grafting increased the tyrosine hydroxylase-expressing and DOPA decarboxylase-expressing neurons in the Nigral and Striatal tissues of rotenone-treated animals

**Fig 4:**
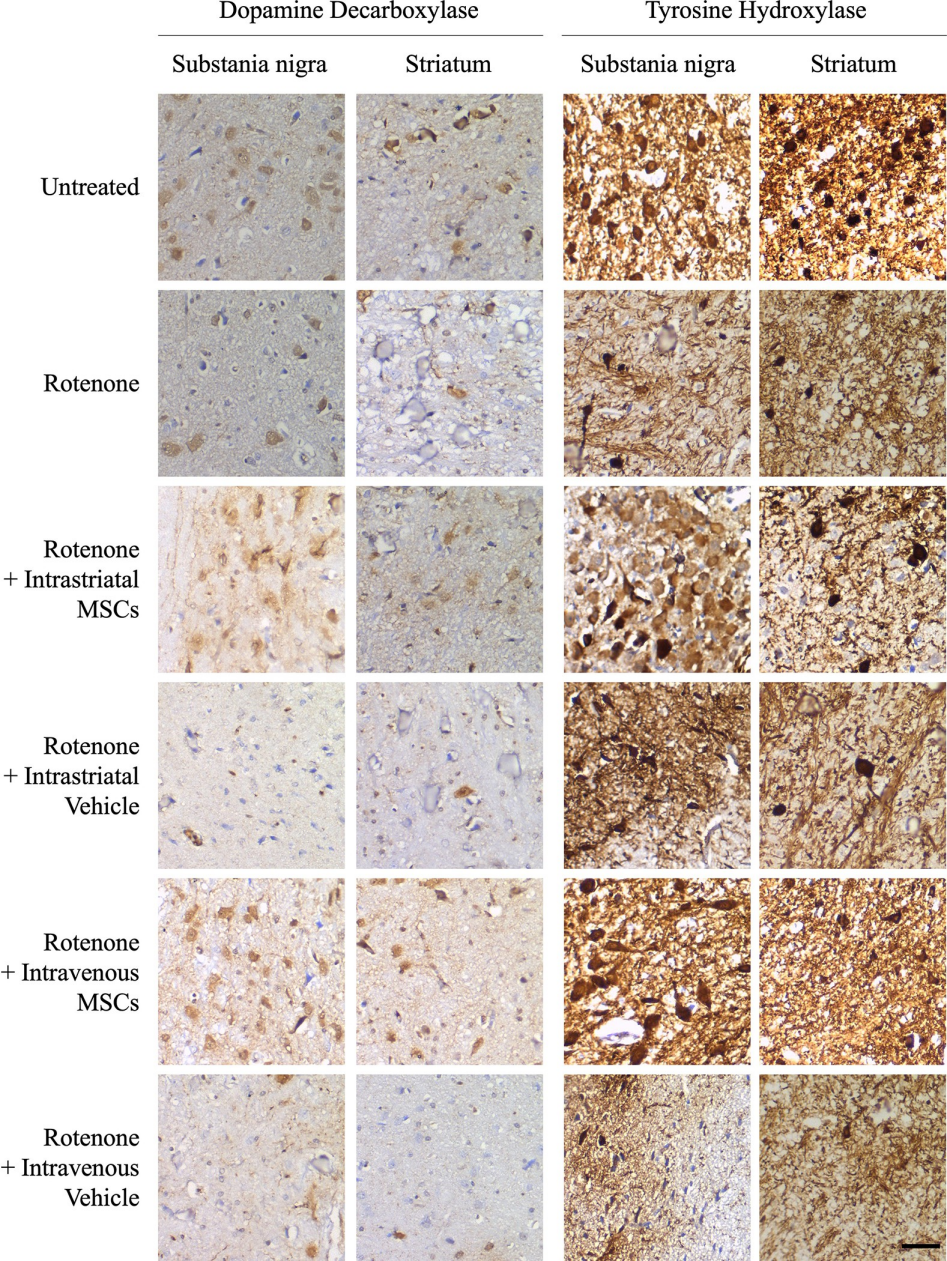
Representative images from brain sections of each group of animals showing immunohistochemistry of endogenous α-Synclein, tyrosine hydroxylase, and Dopa decarboxylase in the Substania Nigra and Striatum. (Bar: 50 μm).

**Fig 5:**
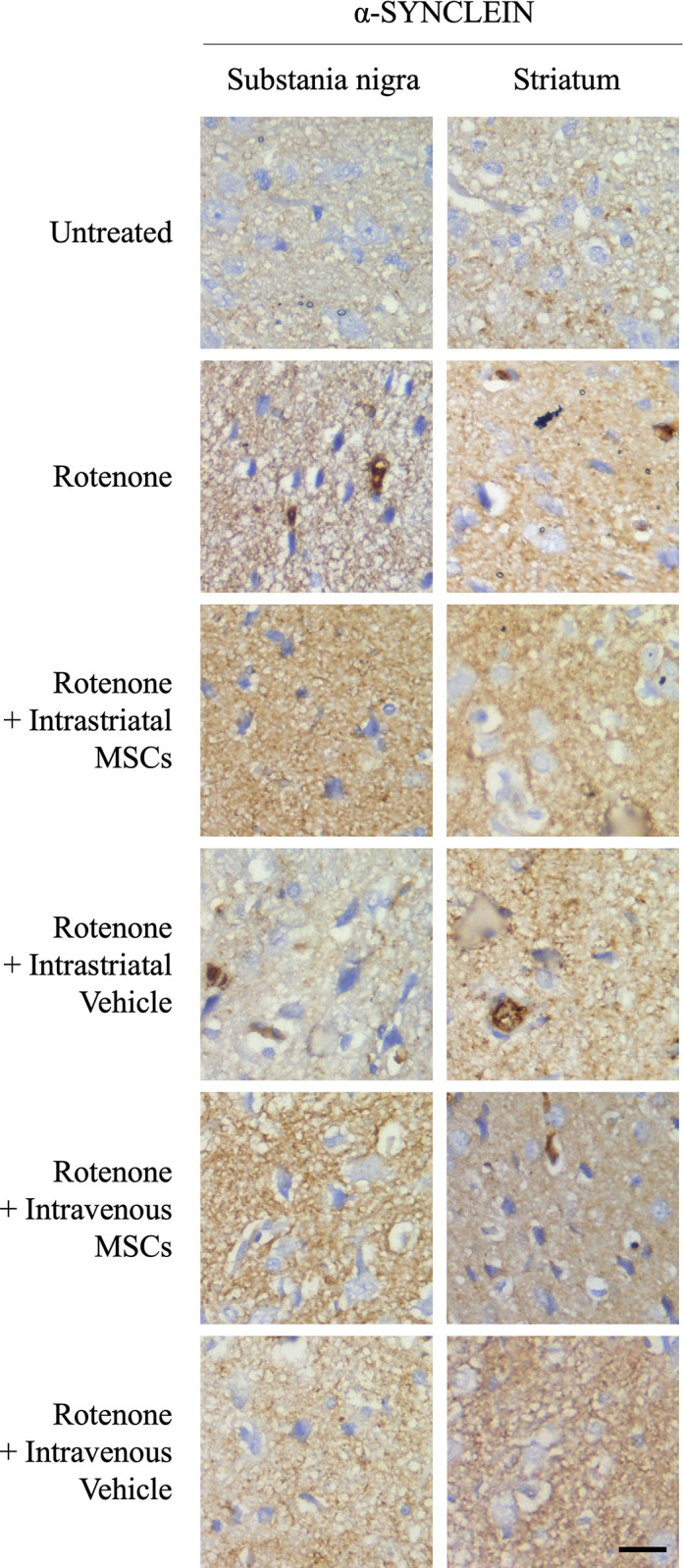
Representative images of each group of animals showing immunohistochemistry of endogenous α-Synclein expression in the Substania Nigra and Striatum. (Bar: 50 μm).

The expression of tyrosine hydroxylase (TH) and DOPA decarboxylase (DDC) within nigral and striatal regions was shown in Fig 4 (untreated group). ROT application resulted in a remarkable down expression of these enzymes. MSCs transplanted animals, either intrastriatally or intravenously, showed an elevated expression of TH and DDC in the neurons. In addition, the ROT-treated animals showed intra-cellular aggregates of α-synuclein positive bodies in some neurons when compared to controls. In MSCs transplanted animals, α-synuclein bodies were not observed in the neurons of SN and ST. Representative images for the experimental groups are in Figs 4 and 5.

### The impact of MSC grafting on the neurochemical parameters in brain tissue

**Fig 6:**
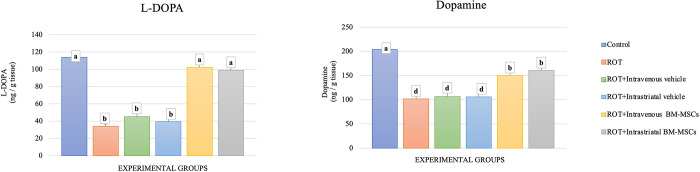
The effect of stem cell administration on l-DOPA and dopamine levels in brain tissue of animals. (ANOVA, n = 4, p < 0.05, Different letters indicate statistically significant differences).

Upon ROT treatment, the levels of DA and l-DOPA in the nigrostriatal tissues of rats were significantly decreased (p < 0.05) when compared to controls. A significant decrease in the levels of both parameters was also observed when the vehicle was applied in combination with ROT as compared to controls. Injection of BM-MSCs significantly elevated (p < 0.05) the levels of DA and l-DOPA in the nigrostriatal tissues as compared to ROT-treated animals. Bar graphs showing these results are placed in Fig 6.

### Markers of oxidative stress

**Fig 7:**
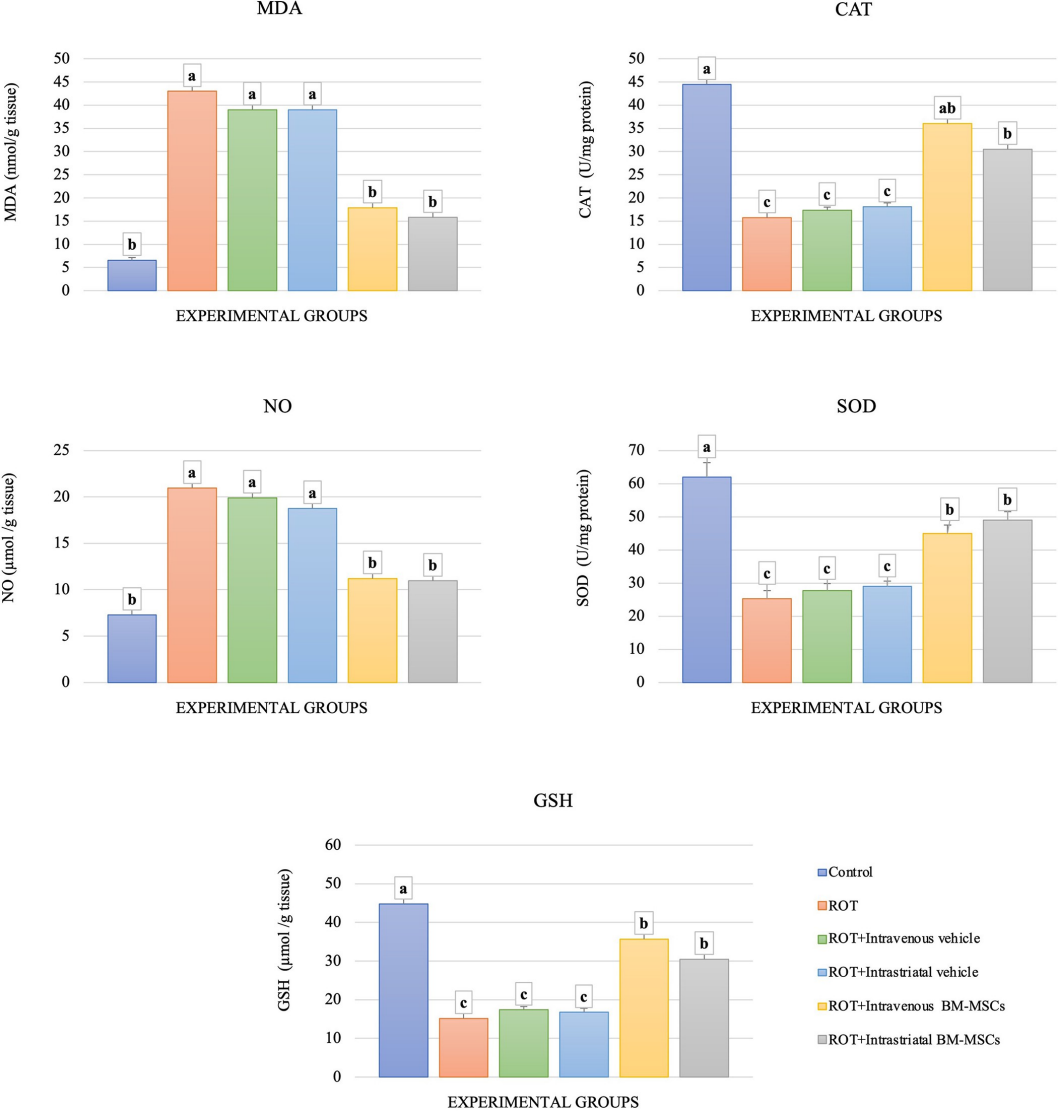
The impact of stem cell administration on the oxidative stress level in brain tissue. (ANOVA, n = 4, p < 0.05, Different letters indicate statistically significant differences).

The brain MDA and NO levels of ROT-treated rats were found to be significantly increased by 6.6 and 2.8 folds, respectively, when compared with the control rats. The application of the vehicle with ROT did not significantly alter these values. However, BM-MSCs transplantation in ROT-treated animals resulted in significantly lowered MDA and NO levels as compared to ROT-treated animals. These values were slightly higher (non-significant difference) than the control values. On the other hand, the activities of SOD and CAT, and the level of GSH were significantly decreased upon ROT application (with or without the vehicle). When applying the BM-MSCs, the levels of these parameters were significantly increased as compared to ROT-treated animals. Bar-graphs showing these results are placed in Fig 7.

### Levels of inflammatory cytokines

**Fig 8:**
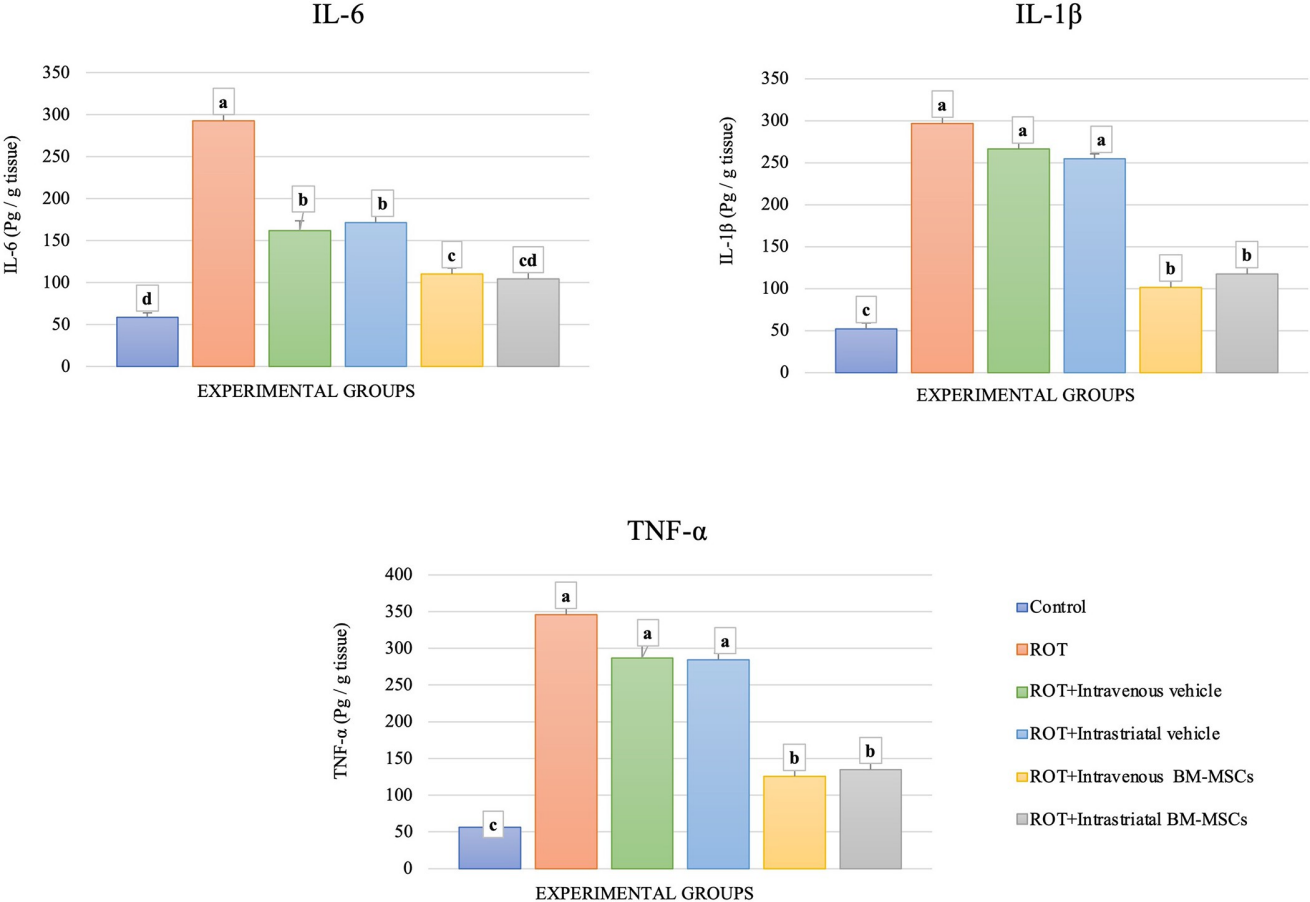
Mesenchymal stem cell administration affects the levels of proinflammatory cytokines. The levels of IL-6 for groups with the ordinary course of inflammation and inflammation on the background of MSCs. (ANOVA, n = 4, p < 0.05, Different letters indicate statistically significant differences).

Regarding the inflammatory response of animals to ROT treatment (with or without the vehicle), the brain levels of IL6, IL1-β, and TNF-α were significantly increased as compared to controls. When applying the BM-MSCs, the levels of these parameters were significantly lowered when compared to ROT-treated animals. Bar graphs showing these results are placed in Fig 8.

## Discussion

PD is one of the most prevalent neurodegenerative disease‎s. The main pathological characteristics of PD include the selective death of mesencephalic nigral dopaminergic neurons and the presence of Lewy bodies in the neurons of the SN [6, 27].

Cellular-based therapy for PD has been previously proposed as a promising treatment strategy [28]. However, the limited feasibility of cellular transplantation renders this approach unsuitable for clinical applications [29]. Intralesional injection of cells appears to be the preferable experimental approach for cell delivery. Stereotaxis-aided injection is usually applied to transplant the cells into the corpus striatum [30]. However, despite the high precision of this method, it requires complex surgical procedures that limit the clinical application for PD cases. On the other hand, the systemic delivery of cells has not been proven to be effective in PD, and the positive results of some studies seem to be due to growth factor upregulation rather than regenerative influence imparted by the transplanted cells [31]. Therefore, this work aims to investigate and compare the therapeutic impact of MSCs transplantation using the previously mentioned routes of delivery on the rotenone-induced PD model in rats.

In this study, rat-derived BM-MSCs were engrafted into ROT-induced rat model of parkinsonism. To evaluate the therapeutic efficiency of MSCs transplantation, brains were evaluated histologically for pathological alternations upon treatments. Neurobehavioral tests (such as the open field and parallel rotarod tests) were carried out. Also, histopathological evaluation of brain sections through immunostaining against TH and DDC (enzymes contributing to the synthesis of dopamine in dopaminergic neurons) was performed. The impact of the BM-MSC grafting was further confirmed by a deep neurophysiological investigation.

Behavioral evaluation of animals assists in studying the symptom-relieving impact of MSC grafting, as behavioral outcomes can be translated into clinical performance in patients. In the current study, ROT application limited the motor abilities of animals. The observed prolonged latency to start moving, increased number of crossed squares, and the number of grooming as well as the decreased number of rears indicate diminished exploratory and locomotor activities in ROT-only treated animals. These changes suggest that ROT administration induces features of P arkinsonism [32]. MSC delivery to the ROT-treated animal model of PD improved all affected neurobehavioral parameters and reversed the ROT-induced behavioral consequences. A previous study mentioned that BM-MSC transplantation can significantly reduce the behavioral abnormalities of rats treated with Quinolinic acid during the six weeks after engraftment [33]. Otherwise, the present behavior findings were in line with other results based on striatal and intravenous transplantation showed that BM-MSCs transplantation significantly alleviates the behavioral impairments resulted from induced dopaminergic neurodegeneration in intrastriatal and intravenous BM-MSCs transplanted groups [34–36]. This alleviation is evidenced by improving the locomotor, and exploratory behaviors in OPT test. Similarly, in Rota-rod test, BM-MSCs transplantation significantly improved rats’ performance, suggesting the role of BM-MSCs in the treatment of PD. However, our results of both tests showed that intrastriatal route was more effective than intravenous route. These findings suggested that both routes of BM-MSCs administration are effective and recommended for PD. Our results were also in agreement with other studies stand on BM-MSCs therapy that showed a significant improvement in rotarod score, recovery of motor activity parameters of OFT, and other behavioral tests in stroke, spinal cord injury, traumatic brain injury, ‎and PD models [37, 38]. Engraftment of MSCs, either intrastriataly [39, 40] or intravenously [36, 41], restored functional DAergic neurons, which in turn improved the behavioral deterioration in different neurotoxins-induced PD models of rats.

We observed that ROT treatment decreased the abundance of dopaminergic neurons in SN and ST. In contrast, injecting MSCs was associated with an increased number of neurons in both regions and improved histological architectures of SN and ST of rotenone-treated animals. This observation is accompanied by a restoration of ROT-induced suppression of the l-DOPA and dopamine levels in the brain in response to BM-MSC engrafting. A previous work mentioned that MSCs were able to be induced into dopaminergic neurons and contribute to PD recovery [42, 43]. Also, BM-MSCs were found to promote cellular proliferation and neurotrophic function of Schwann cells *in vivo* [44]. These observations confirm the previously reported therapeutic potential of MSC xenografting in PD models [45].

Importantly, our study has focused on the regenerative ability of BM-MSCs and their ability to target sites of inflammation and damaged areas for assisting ‎in the recovery and repair of dysfunctional DAergic neurons ‎in SN and ST regions. Therefore, we labelled the two most important markers for the dopaminergic neurons, TH and DDC, in brain tissues. ROT application dramatically decreased the number of TH and DDC positive cells and increased the frequency of intracellular α-synuclein positive bodies in the SN and ST regions as compared to controls. MSCs grafting restored the TH and DDC positive cells in these regions accompanied by lower count of α-synuclein positive bodies when compared to the ROT-treated animals. TH and DDC are markers for dopamine-producing cells in the brain. TH catalyzes the conversion of Tyrosine into l-DOPA and is considered the rate-limiting enzyme in dopamine production [46]. DDC is responsible for the synthesis of dopamine from l-DOPA [47]. Similarly, the work of Chi and colleagues observed that MSC infusions into 6-OHDA-lesioned animals showed more TH-positive cells compared with the 6-OHDA-only group [48]. Another work showed that rats with MSC grafting displayed significant preservation in TH-positive fibers in the ST and the number of TH-positive neurons in SN compared to that of control rats [43]. Another recent work showed that the basic cellular phenotype of injected MSCs changed to cells with neural morphology that express specific markers for dopaminergic neurons including TH [40]. Altogether, these observations strongly support the potential role of the xenografting MSCs in regenerating the dopaminergic neurons at damaged areas of the brain. These histopathological outcomes in SN and ST are in accordance with the previously mentioned motor benefits in our behavioral analysis in response to MSC application to rotenone-treated animals. This further confirms the potential therapeutic impact of MSC treatment.

Oxidative stress responses and inflammatory actions in SN are key players in promoting PD. Previous studies showed that the motor impairment caused by ROT administration, has been shown to be result from selective degeneration and loss of DA neuronal cells, that is at least in part correlated to inflammatory actions and oxidative stress [49, 50]. Our study showed that the transplantation of BM-MSCs lowered the levels of oxidative stress and nitrosative impact induced by ROT. This effect was observed as a significant reduction of MDA and NO levels in nigrostriatal tissues and suggests a possible role of BM-MSCs in minimizing the level of free radicals. A possible explanation is that the injected MSCs have possibly improved the mitochondrial function in nigrostriatal cells and subsequently improved the metabolic processes in these cells [51]. By comparing the results of two routes of BM-MSCs administration, we found that the results of NO and MDA parameters in intrastriatal group were better than the results of intravenous group. In line with previous studies, the present work showed a significant decrease in antioxidant enzyme activities (SOD and CAT) concurrently with a significant decrease in GSH level in the nigrostriatal tissue of ROT and sham-grafted groups. A decline in the level of these parameters results in a loss of tolerance to oxidative damage [52]. This perturbation of antioxidant defense system was likely initiated to reduce the continuous injurious consequences of overrun oxidative stress and possible mitochondrial dysfunction resulting from ROT toxicity [53, 54]. A weakened antioxidants system such as SOD, CAT, and GSH has been well authenticated in the PD brain [50, 55]. Hasan et al. (2019) [56] stated that ROT exposure ‎promotes‎ ROS production in the mitochondria ‎of striatal cells that is associated with the reduction of GSH, CAT, and SOD. Similarly, Tseng et al. (2020) observed that ROT increased striatal nitric oxide and thiobarbituric acid reactive substance (TBARS) levels and decline in GSH, CAT, and SOD levels, which suggested that both NO and free radicals may participate in the motor impairment development. These findings suggested that both routes of BM-MSCs administration are effective and recommended for PD, and likely is one of the mechanisms by which the oxidative and nitrosative injuries are alleviated, and metabolism processes are improved with these treatment protocols. On the other hand, SOD and CAT activities and GSH level were, at least partially, restored upon MSC application in the ROT-treated rats. It is well known that these proteins are considered the primary defense line against the damage of ROS. Therefore, infusion of MSCs had possibly alleviated and repaired damaged cells by neutralization of ROS and lowering the level of oxidative stress [14, 57].

Inflammation is one of the most important events initiated in response to oxidative stress in animals. Therefore, we followed the inflammatory responses in the brains of animals. In this work, BM-MSC injection significantly suppressed the elevated the ROT-induced IL-6, ‎IL-1β, and TNF-α levels in the nigrostriatal tissue. This supports a potential immunomodulatory role of BM-MSCs, and the ability of BM-MSCs to reduce the continuous activation of microglia, that in turn not only diminishes the release of pro-inflammatory cytokines but also reduces oxidative and nitrosative stress and improves the potential of antioxidants [58]. These cellular abnormalities lead to the loss of DA neurons, decreased DA production, and subsequently motor alterations [59]. Also, Nakajima and co-workers showed that intravenous administration of MSCs resulted in a significant improvement in posture score and Rota-rod performance, and suppresses the expression of various pro-inflammatory cytokines in the brain tissue, such as TNF-α, IFN-γ, IL-6, IL-1β, and IL-2 in the inflammatory environment compared with vehicle [60]. The work of Rossignol and colleagues demonstrated that after xenotransplantation of human MSC into rat brain, there were higher levels of messenger RNAs for the anti-inflammatory molecules IL-6 and TGF-β1 than for other pro-inflammatory cytokines [61]. In accordance, the IV transplantation of MSCs causes a significant increase in the anti-inflammatory cytokine IL-10 levels sided with a reduction in the level of pro-inflammatory cytokines TNF-α, IL-1β, ‎IL-6, and IFN-γ [62].

In conclusion, the present study identified the positive effects of intrastriatal and intravenous delivery of MSCs in a progressive rat model of PD. The observed beneficial effects in improving neurobehavioral changes, alleviating oxidative stress and neuroinflammation, reforming antioxidants potential, and restoring the function of DAergic neurons in nigrostriatal tissues of the brain suggest potential benefits in clinical applications. Notably, the absence of significant disparities in treatment outcomes between the two delivery methods suggests a comparable efficacy, albeit with a notable distinction in safety and feasibility. The intravenous route emerges as a seemingly safer and more feasible option when weighed against the intrastriatal approach. Also, future investigation is necessary prior to clinical application to further assess other parameters such as the delivery vehicles, number of injected cells, and investigation of the ideal type of stem cells and timing of transplantation.

## Supporting information

S1 Data The attached supporting file contains the raw data sets regarding the neurochemical measurements and the behavioral tests. (XLSX)
